# A next-generation beneficial microbe: *Akkermansia muciniphila*

**DOI:** 10.3164/jcbn.18-57

**Published:** 2018-06-20

**Authors:** Yuji Naito, Kazuhiko Uchiyama, Tomohisa Takagi

**Affiliations:** 1 Molecular Gastroenterology and Hepatology, University Hospital, Kyoto Prefectural University of Medicine, 465 Kajii-cho, Kamigyo-ku, Kyoto 602-8566, Japan; 2 Department of Endoscopy and Ultrasound Medicine, University Hospital, Kyoto Prefectural University of Medicine, 465 Kajii-cho, Kamigyo-ku, Kyoto 602-8566, Japan

**Keywords:** *Akkermansia muciniphila*, diabetes, polyphenols, cancer immunotherapy

## Abstract

There have been many reports on the roles of intestinal flora and intestinal environment in health promotion and disease prevention. Beneficial bacteria such as *Bifidobacterium* and lactic acid-producing bacteria have been shown to improve the intestinal environment, and yield a good effect on metabolism, immunity and nerve response. In this review, in addition to these beneficial bacteria, we introduced *Akkermansia muciniphila* as a next-generation beneficial microbe. Several reports indicate that *Akkermansia muciniphila* affects glucose metabolism, lipid metabolism, and intestinal immunity, and that certain food ingredients such as polyphenols may increase the abundance of *Akkermansia muciniphila* in the gut.

## Introduction

In 2004, Muriel Derrien in her Ph.D. research at Wageningen University of the Netherlands isolated from a sample of healthy human feces a species of bacteria that can grow on a viscogenic substrate such as mucin and use it a single nutrient source, especially on the mucosal surface of the gastrointestinal tract.^(1)^ From the name of microbial ecologist Antoon DL Akkermans and 6“preferring mucin”, this bacterium was named *Akkermansia muciniphila* (*Akkermansia*). It accounts for 1 to 4% of intestinal bacteria in adults and is a species of bacteria that inhabits the large intestine.^(2)^ *Akkermansia a* is a gram-negative, obligate anaerobic, non-motile, nonspore-forming elliptical eubacterium, classified under the phylum Verrucomicrobia. In this review, we summarized recent studies that have indicated that *Akkermansia* is involved in obesity, glucose metabolism, and intestinal immunity, as well as reports on the associated role of food factors.^(3,4)^

## Glucose Metabolism and *Akkermansia*

In humans with high body weight, body mass index (BMI), blood cholesterol level, and fasting blood glucose level, it is suggested that the abundance of *Akkermansia* in the gut is lower than that in the gut of healthy humans.^(5)^ In addition, when overweight or obese people undergo calorie-restricted diet therapy, the effect of improving insulin resistance has been reported to be more pronounced in humans with a higher abundance of *Akkermansia* in the intestine. It is reported that *Akkermansia* increases when metformin, which is one of the therapeutic agents for diabetes, is administered to obese mice, and the action of metformin is partly mediated by the action of *Akkermansia*.^(6)^ In recent years, it has been revealed that, in the diabetic state, the breakdown of the intestinal mucosal barrier mechanism modifies the pathological condition; it has been reported that *Akkermansia* promotes mucus secretion and makes the barrier mechanism more robust. Chelakkot *et al.*^(7)^ demonstrated that *Akkermansia*-derived extracellular vesicles may act as functional moieties for controlling gut permeability and that the regulation of intestinal barrier integrity can improve metabolic functions in high-fat diet-fed mice. Blood lipopolysaccharide (LPS) concentration, an indicator of intestinal permeability, was also observed to be high in obese subjects (high-fat diet and diabetes mellitus mouse models), and the administration of* Akkermansia *was shown to decrease it.^(8)^**In mice, many studies have been carried out towards showing how *Akkermansia* more directly influences glucose/lipid metabolism.

The precise molecular mechanisms underlying how *Akkermansia* physiologically influences the human body are gradually being elucidated. It is thought that *Akkermansia* produces short-chain fatty acids such as acetic acid from mucin and supplies energy to goblet cells that produce mucin. Metformin, an antidiabetic drug, is suggested to increase the number of goblet cells, thereby enhancing mucin production, thickening the intestinal mucus layer, and maintaining the intestinal barrier mechanism; this contributes to an anti-inflammatory effect and, consequently, its antidiabetic action.^(6)^ Studies analyzing the bacterial cell proteins of *Akkermansia *have also been carried out. Amuc-1100, an outer membrane protein of *Akkermansia*, has been identified and found to activate intracellular signals mediated by the Toll-like receptor 2 (TLR2) of intestinal epithelial cells, contributing to the enhancement of the intestinal barrier.^(9)^ It has also been demonstrated that Amuc-1100 of *Akkermansia* is involved in the immune response, specifically in the induction of the production of interleukin-10 (IL-10), which is an anti-inflammatory cytokine.^(9)^ As previously mentioned, it has become clear that *Akkermansia* is either directly or indirectly involved in the metabolic and immune responses of humans, thus attracting attention as a next-generation beneficial bacterium.^(3)^

## Polyphenol Functionality and *Akkermansia*

The health-promoting and disease-preventing effects of polyphenols have attracted attention. There are many polyphenols in nature, including catechins found in wine, tea, apples, grape leather, mussels and blueberries; isoflavones found in soybeans; and chlorogenic acid found in coffee. Polyphenols generally have low absorption rates, and there previously suggested to not work effectively in the body. However, it has recently been reported that polyphenols are metabolized by intestinal bacteria to change its absorption rate and bioavailability; conversely, polyphenols can change the composition of the intestinal bacterial bacteria. Intestinal bacteria that degrade polyphenols such as quercetin have been reported, and the relationship between polyphenols and the gut microbiota is becoming an important subject for the evaluation of food functionality.

Polyphenols derived from grapes act to increase the abundance of *Akkermansia* in the intestinal tract; as a result, they have been shown to enhance intestinal barrier function and incretin secretion from intestinal endocrine cells.^(10,11)^ Polyphenols derived from cranberries have also been reported to increase the abundance of *Akkermansia*, as well as help suppress obesity, insulin resistance, and intestinal inflammation.^(12)^ These indicate that there is an additional pathway that exerts its function by acting on the gastrointestinal tract without absorption of polyphenols, by influencing the intestinal bacterial flora and acting on intestinal mucosal cells. Masumoto *et al.*^(13)^ demonstrated that apple-derived macromolecular procyanidins induce an increase in the abundance of intestinal *Akkermansia* and have anti-inflammatory and anti-metabolism effects in a mouse model of metabolic syndrome. Administration of macromolecular procyanidins suppressed changes in Inflammation in intestinal mucosa, weight gain, and abnormalities in liver lipid metabolism induced by a high-fat high-sucrose diet. In addition, 16S rRNA metagenomic analysis showed an improvement in the Firmicutes/Bacteroidetes ratio, as well as an increase in *Akkermansia*, leading to the anti-inflammatory action in the intestinal tract and the enhancement of the intestinal barrier function.

As far as the functionality of polyphenols has been deemed to contribute to the strong antioxidant effect observed in *in vitro* experiments, recent results have indicated that relatively poorly absorptive polyphenols directly affect intestinal bacteria and show the possibility that the antioxidant effect is mediated by so-called “good bacteria” such as *Akkermansia*. However, polyphenols have not been sufficiently analyzed in human subjects, and the evidence for its involvement in increasing the abundance of *Akkermansia* has not been sufficient.

## Cancer Immunotherapy and* Akkermansia*

Therapeutic efficacy and its association with the intestinal microbiota were analyzed in 249 patients with lung, kidney, or bladder cancers treated with immune checkpoint inhibitors.^(14)^ As a result, patients who used antibiotics before and after treatment had a poorer response to the immune checkpoint inhibitor PD-1 antibody and a shorter survival time compared to the patients who did not use antibiotics. In addition, in the intestine of a patient who responded positively to the immune checkpoint inhibitor, an increase in the abundance of *Akkermansia* was observed compared to a patient who did not respond positively to the immunotherapy. It was also confirmed that, using the stool of a patient that responded positively to the immunotherapy, fecal microbiome transplantation in a sterile mouse caused it to respond positively to the anti-PD-1 antibody. In future cancer immunotherapy, we should obtain detailed genetic information on the cancer tissue, identify the mutation of the mismatch repair gene, select an immune checkpoint inhibitor based on this information, and refer to metagenomic information on the gut microbiota of the patient.

## *Akkermansia* in Japanese

**Fig. 1:**
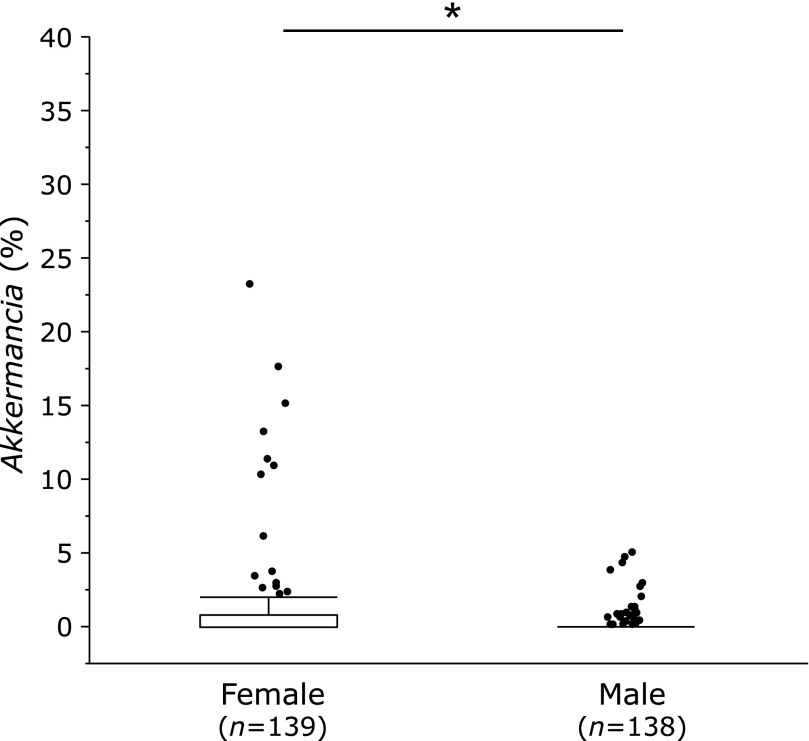
The relative abundance of *Akkermansia miciniphila* in female and male subjects. Microbiota in fecal samples were analyzes by 16S rRNA V3–V4 gene sequencing. *p<0.05.

Recently, we conducted a 16S rRNA V3–V4 sequence analysis on the feces microbiota of about 300 Japanese people.^(15)^ Interestingly, the relative abundance of *Akkermansia *in female was significantly higher than that in male (Fig. 1), however, the proportion of this bacteria being present at 5% or more was extremely low at 3.6% of females, which was similar to the previous report.^(16)^ It is an important task for the future to study what kinds of meals and exercises can increase* Akkermansia* and to apply it to Japanese people.

## Conclusion

In addition to traditional beneficial bacteria such as *Bifidobacterium* and lactic acid-producing bacteria, we introduced the next-generation “beneficial bacteria” called *Akkermansia muciniphila*. It is considered as a target of the functionality of polyphenols as well as cancer immunotherapy, and it is important to verify its effect in human clinical trials in the future.

## Author Contributions

YN, TT and KU were involved in editing the manuscript. All authors discussed the results and commented on the manuscript.
